# The unmatched

**Published:** 2018-05-31

**Authors:** Amit Persad

**Affiliations:** 1 Division of Neurosurgery, University of Saskatchewan, Saskatchewan, Canada

On March 1, 2017, just over a year ago, like all other Canadian medical students participating in CaRMS, I woke up nervous but eager to find out where I would be spending the next phase of my medical training. I had avoided thinking about CaRMS ever since submitting my rank list, but the stress of the situation became more pressing with each passing minute. With only a half hour left before results were released, there was hardly anything else on my mind. I remember being in my car at 10am, pulling over into a grocery parking lot to open the result page. My phone was being bombarded with messages from family members, friends, and classmates sharing their results and inquiring about my own.

*We regret to inform you that you did not match to a position*.

My initial reaction was shock. I could not believe that this had been my outcome. I had done everything they told me was needed, like so many other candidates before me who had been in the same position. I initially did not believe it, and had to take a moment where I just sat there in the car. I must have refreshed the page at least twenty times, waiting for the error to correct itself or to reveal itself to be a joke. The email from the University of Alberta Undergraduate Medical Education office requesting my presence at an urgent planning meeting cemented the crushing reality to me.

Though I had recognized the possibility of being unmatched, I did not dwell on the chance. Like many other students in my situation, I settled myself with the fact that I had worked hard on my rotations, read diligently, and done electives and research in order to strengthen my application. There was a fair amount of entitlement inherent in this attitude, and I was not exempt from this. The difficulty lies in numbers: when too many students apply to a specialty, some must go unmatched.

There are many studies that try to identify what makes a good resident.^1,2^ Factors like academic inclination, clinical reviews, reference letters, personal statements, and interviews are all taken into account by the reviewing body and used to make a gestalt of each individual, which is then tested by the interview process. It is truly a subjective process that is imperfect and based on tenets that are unclear to both the candidate and the program.^3^ Ongoing discussion related to Wilson & Bordman^3^ reflects a lack of clarity and a recognition that the process needs to be improved, with a marked lack of objective criteria.^4^ Programs do not know what to look for in a candidate, sometimes asking silly questions at interviews and making decisions that are life changing for applicants, as suggested to me by a program director.

It is impossible to ignore that this system does not work for everyone. 2017 set a record for the number of unmatched students in Canada.^5^ One student unfortunately took his own life after being left without a residency position for two consecutive years, leaving behind a detailed note of his grievances with the system. Typically, neurosurgery is a discipline with a similar number of applicants and available positions. The year I entered CaRMS did not fit that pattern, with 31 qualified applicants applying to only 15 positions. Many individuals who may have made excellent residents and eventually neurosurgeons were not selected for admission to residency due, almost entirely, to sheer numbers.

For me, the condition of being unmatched was devastating. I recall sitting through the emergency meeting with our faculty counselors and not looking up from the floor. I could not believe the situation I was in. Two months later, I remember writing my MCCQE Part 1 exam and frequently being tempted to change answers that I was sure that I had answered correctly, simply because I was no longer as confident in myself. I approached many of the neurosurgery faculty that I trusted for advice, which was largely the same: CaRMS usually does not work the second time;^6^ being unmatched would almost inevitably lead to practice outside of neurosurgery. Even surgeons who had given me glowing evaluations and thought that I would be a highly sought-after candidate were not optimistic at my chances a second time. Their reversal of opinion was earth-shattering.

Interestingly and fortunately, as I continued to seek out opinions, some residents and faculty opened up to me about their difficulties with matching. A well-known faculty member I completed an elective with shared with me his experience of being unmatched, but being able to get an empty spot in the second round. We agreed that it was a confidence-breaking situation that felt frightening and hopeless. However, now I knew that there were those who had shared my experience. This was a faculty member that I had worked closely with and respected, and I was encouraged by the similarity of his experience.

Another faculty member in cardiac surgery, with whom I had worked during a selective, also reached out to me sharing his experience. He had not initially matched to his specialty of choice; instead of cardiac surgery he was matched to general surgery. He had come to enjoy his general surgery residency, but a position eventually opened up in his third year of residency and he was able to switch over. His advice was that even if you don’t match to your first choice specialty, there are often routes to return to it. Even though he eventually switched, he encouraged me to give the discipline that you are matched to a chance.

The connections that I made with people, such as these, while being unmatched were helpful to me and gave me a lot of insight into the way that they handled themselves during their period of not matching. Without exception, all had reflected on themselves and where they might have gone wrong, and had become more grateful for their position after their experience. All were motivated to securing a position in their disciplines of choice after securing a spot in a different residency, and eventually became highly successful in the field of their dreams.

I also had the opportunity to talk to a few residents in other disciplines who had not matched to neurosurgery, but had wanted to. I asked them about their own experiences. Most of them were bitter about CaRMS and toward neurosurgery as a discipline. Moreover, they were somewhat disparaging of many of their colleagues who had matched to neurosurgery. In some cases, previously unmatched candidates were quite acidic, particularly regarding the individuals who had left the specialty. In their eyes, this was a waste of precious neurosurgical positions. Certainly, I understand where these individuals’ feelings stem from, but this animosity neither makes one a stronger individual nor one suited to train in a program. In fact, it may subvert the learning that can come from being unmatched.

The experience of being unmatched was certainly negative. In fact, it was likely the worst thing that has ever happened to me. But what I want to communicate is that the experience, *because* it was the worst thing that happened to me, was good. One of the most important abilities of a resident physician is the ability to cope with stress.^1^ A prior study on attitudes of staff neurosurgeons towards incoming neurosurgery residents is that the ability to handle stress is a skill that is more difficult to teach and more important to have when entering. I did not know how I would handle failure beforehand, but now I know that I am able to tolerate and recover from failure related stress relatively effectively.

In addition, I was met with a situation that had come about in large part due to my actions and choices. Many poor outcomes in medicine can be written off as circumstantial or multifactorial, and in a lot of cases this is certainly true. Even in my case, it would be easy to attribute my lack of success to numbers and circumstances (15 spots and 31 applicants). Again, this is likely partially true, but to ignore the role that I played in my failure to match would be to miss out on the learning that came from the subsequent journey.

Many people applying to medical residencies will have the same set of experiences that I did beforehand, with no real exposure to failure. A lifelong string of uninterrupted success fosters a quiet sense of entitlement, which is sustained with each consecutive achievement. This is not necessarily an active sentiment, but rather one that comes from continual success. For me, it was a transformative experience. I knew that I could not rest on my laurels and simply re-apply again with a similar application; it did not work the first time, why would it work again? I would need to be the best that I could be, and that attitude translated into the rest of my work. I gave a lot more of my effort to the tasks before me, from clinical work to publications. I decided that I would try again; I took the mindset that if every neurosurgeon who failed something along the way gave up, there would be none practicing.

Being unmatched in CaRMS created the potential for immense growth. For me, this took the form of no longer taking success for granted, increasing my work ethic, and learning to cope with stress and uncertainty. This growth may look and feel differently for others. My aim was not to point out the flaws in CaRMS, or to critique resident selection, but to emphasize to students and programs that those applicants who have endured an unmatched cycle are not necessarily the same people they were when they moved through the process of electives and interviews the first time. The experience of going unmatched can elicit profound changes and personal growth through intense introspection. To other students in this same position as I was, I am not saying it is by any means a desirable journey one should long for, but it is an opportunity for reflection and improvement. And it could become a marker of desirable residency candidates. Jordan Peterson wrote in his book *12 Rules for Life:* “There are so many ways that things can fall apart, or fail to work altogether, and it is always wounded people who are holding it together. They deserve some genuine and heartfelt admiration for that. It is an ongoing miracle of fortitude and perseverance”.^7^ This was in reference to those ill or wounded, but for the candidates recovering from the shock of an unsuccessful match it may have some relevance as well. To move forward shows a fortitude that would serve the candidate well in a gruelling residency.

I have been granted a position with a neurosurgery program only four months after being unmatched. However, those are four months that I will never forget and have taught me lessons that I hope will benefit me throughout a challenging residency. My experience has left me with an openness to learn, an ability to recognize my limitations and areas for growth, and a sense of gratitude to be able to work in the field that I fell in love with. To any of my colleagues and future colleagues enduring the hardships of an unmatched cycle—try your hardest to find opportunity buried in disappointment, replace your fear, self-doubt, and hopelessness with determination and humility, and you will emerge a stronger CaRMS applicant.

## References

[1] Myles ST, McAleer S Selection of neurosurgical trainees. Can J Neurol Sci. 2003;30(1):26-30. 12619780 10.1017/s0317167100002390

[2] Lipsman N, Khan O, Kulkarni AV “The Actualized Neurosurgeon”: A Proposed Model of Surgical Resident Development. World Neurosurg. 2017;99:381-6. 28012887 10.1016/j.wneu.2016.12.039

[3] Wilson CR, Bordman ZN What to do about the Canadian Resident Matching Service. CMAJ. 2017;189(47):E1436-7. 29180382 10.1503/cmaj.170791 PMC5703675

[4] Persad A The overall culture of residency selection needs fixing. CMAJ. 2018 Apri 9; 190(14):E443 29632041 10.1503/cmaj.68993 PMC5893322

[5] Vogel L Record number of unmatched medical graduates. CMAJ. 2017;189(21):E758-9. 28554957 10.1503/cmaj.1095432 PMC5449246

[6] Bumsted T, Schneider BN, Deiorio NM Considerations for Medical Students and Advisors After an Unsuccessful Match. Acad Med. 2017;92(7):918-22. 28328737 10.1097/ACM.0000000000001672

[7] Peterson J 123 Rules for Life: An Antidote to Chaos. 2018 Penguin Random House ISBN 978-0-345-81602-3.

